# Draft Genome Sequence of the Lignocellulolytic and Thermophilic Bacterium Thermobacillus xylanilyticus XE

**DOI:** 10.1128/mra.00934-21

**Published:** 2022-03-08

**Authors:** Harivony Rakotoarivonina, Valentin Loux, Christelle Doliwa, Véronique Martin, Caroline Rémond

**Affiliations:** a Université de Reims Champagne Ardenne, INRAE, FARE, UMR A 614, Chaire AFERE, Reims, France; b Université Paris-Saclay, INRAE, MaIAGE, Jouy-en-Josas, France; c Université Paris-Saclay, INRAE, BioinfOmics, MIGALE Bioinformatics Facility, Jouy-en-Josas, France; University of Southern California

## Abstract

Thermobacillus xylanilyticus is a thermophilic and hemicellulolytic bacterium able to use several lignocelluloses as its main carbon source. This draft genome sequence gives insight into the genomic potential of this bacterium and provides new resources to understand the enzymatic mechanisms used by the bacterium during lignocellulose degradation and will allow the identification of robust lignocellulolytic enzymes.

## ANNOUNCEMENT

Thermophilic microorganisms may provide robust and thermostable enzymes for plant biorefining to produce biofuels and biomolecules (1, 2). Thermobacillus xylanilyticus XE (deposited at the Collection Nationale de Cultures Microbiennes France under no. CNCM I-1017) is a Gram-positive, thermophilic bacterium, originally isolated from farm soil situated underneath a manure heap, that is able to use nonpretreated lignocelluloses as its carbon source (3–5). This bacterium produces several hemicellulases (xylanase, arabinosidase, and feruloyl esterase) that have resistance to high temperatures (60° to 70°C) and alkaline reaction conditions (4–10) and efficiently liberate sugars and phenolic compounds from various lignocelluloses (8, 10–15).

*T. xylanilyticus* was grown at 50°C on basal medium complemented with 5 g/L xylan (3). The total genome was isolated from early-stationary-phase cell cultures using the Purelink genomic DNA kit (Thermo Fisher). DNA was quantified using a fluorimetry methodology (Qubit v2.0; Thermo Fisher) and by gel electrophoresis analysis. Genomic DNA was fragmented with a Covaris S220 instrument. DNA libraries were prepared with a TruSeq SBS kit v5 (Illumina) protocol and the SPRIworks fragment library system I. The genome was sequenced by using 74-nucleotide paired-end sequencing with Genome Analyzer IIx instruments (Illumina). Raw read quality was assessed using FastQC (http://www.bioinformatics.babraham.ac.uk/projects/fastqc/), and quality filtering was performed using Sickle (https://github.com/najoshi/sickle). The genome sequence was assembled *de novo* using Velvet v1.1. 04 (16) after quality analyses and trimming of raw reads. The annotation was performed with the AGMIAL platform (17). Default parameters were used for all software.

A total of 32,786,406 raw reads were obtained, representing an average coverage of 58×; 240 Mb of raw sequences was assembled into 108 scaffolds (146 contigs). The *N*_50_ value of the assembly was 83 kb. The largest contig size was 184,473 bp. The size of the genome was 4,109,925 nucleotides with a G+C content of 61.0%. The draft genome encoded 3,956 proteins and 55 tRNAs; 1 copy each of 23S rRNA and 16S rRNA was correctly assembled. The lignocellulolytic potential of *T. xylanilyticus* was assessed by identifying the relevant genes for lignocellulose utilization (CAZymes) by using a procedure analogous to that used for the analysis of daily releases of GenBank data by the CAZy database (www.cazy.org) (18) and involving both automated and human curation-based BLASTP (https://blast.ncbi.nlm.nih.gov/Blast.cgi) and HMMER v3 (http://hmmer.janelia.org/) results against libraries of modules derived from the database. A total of 94 genes encoding glycoside hydrolases, 16 genes encoding carbohydrate esterases, 45 genes encoding glycosyl transferases, and 2 genes encoding polysaccharide lyases were identified. Some of these genes were organized in operon structures. Except for genes encoding β-glucosidases and one putative gene encoding an exoglucanase, no other genes related to cellulose degradation were identified. Several oxidoreductases have been annotated. This genome sequence will facilitate the identification of new genes implicated in the enzymatic fractionation of lignocellulosic biomass for the production of new enzymes of interest.

### Data availability.

*T. xylanilyticus* raw reads and the draft genome sequence have been deposited at the European Nucleotide Archive (ENA) under the study accession number PRJEB43105. The raw reads are available under run accession number ERR5840465. The annotated genome sequence is available under WGS sequence set CAJRAY010000000. The version described in this paper is the first version.

## References

[1] Bhalla A, Bansal N, Kumar S, Bischoff KM, Sani RK. 2013. Improved lignocellulose conversion to biofuels with thermophilic bacteria and thermostable enzymes. Bioresour Technol 128:751–759. doi:10.1016/j.biortech.2012.10.145. 23246299

[2] Viikari L, Alapuranen M, Puranen T, Vehmaanperae J, Siika-Aho M. 2007. Thermostable enzymes in lignocellulose hydrolysis. Adv Biochem Eng Biotechnol 108:121–145. doi:10.1007/10_2007_065. 17589813

[3] Touzel J-P, O’Donohue M, Debeire P, Samain E, Breton C. 2000. *Thermobacillus xylanilyticus* gen. nov., sp. nov., a new aerobic thermophilic xylan-degrading bacterium isolated from farm soil. Int J Syst Evol Microbiol 50:315–320. doi:10.1099/00207713-50-1-315. 10826818

[4] Rakotoarivonina H, Hermant B, Monthe N, Remond C. 2012. The hemicellulolytic enzyme arsenal of *Thermobacillus xylanilyticus* depends on the composition of biomass used for growth. Microb Cell Fact 11:159. doi:10.1186/1475-2859-11-159. 23241174 PMC3541102

[5] Harris GW, Pickersgill RW, Connerton I, Debeire P, Touzel J-P, Breton C, Perez S. 1997. Structural basis of the properties of an industrially relevant thermophilic xylanase. Proteins 29:77–86. doi:10.1002/(SICI)1097-0134(199709)29:1<77::AID-PROT6>3.0.CO;2-C. 9294868

[6] Connerton I, Cummings N, Harris GW, Debeire P, Breton C. 1999. A single domain thermophilic xylanase can bind insoluble xylan: evidence for surface aromatic clusters. Biochim Biophys Acta 1433:110–121. doi:10.1016/S0167-4838(99)00151-X. 10446364

[7] Debeche T, Cummings N, Connerton I, Debeire P, O’Donohue MJ. 2000. Genetic and biochemical characterization of a highly thermostable alpha-l-arabinofuranosidase from *Thermobacillus xylanilyticus*. Appl Environ Microbiol 66:1734–1736. doi:10.1128/AEM.66.4.1734-1736.2000. 10742272 PMC92053

[8] Rakotoarivonina H, Hermant B, Chabbert B, Touzel J-P, Remond C. 2011. A thermostable feruloyl-esterase from the hemicellulolytic bacterium *Thermobacillus xylanilyticus* releases phenolic acids from non-pretreated plant cell walls. Appl Microbiol Biotechnol 90:541–552. doi:10.1007/s00253-011-3103-z. 21279344

[9] Samain E, Debeire P, Touzel JP. 1997. High level production of a cellulase-free xylanase in glucose-limited fed batch cultures of a thermophilic *Bacillus* strain. J Biotechnol 58:71–78. doi:10.1016/S0168-1656(97)00140-5. 9383981

[10] Rakotoarivonina H, Revol PV, Aubry N, Remond C. 2016. The use of thermostable bacterial hemicellulases improves the conversion of lignocellulosic biomass to valuable molecules. Appl Microbiol Biotechnol 100:7577–7590. doi:10.1007/s00253-016-7562-0. 27142296

[11] Remond C, Aubry N, Cronier D, Noel S, Martel F, Roge B, Rakotoarivonina H, Debeire P, Chabbert B. 2010. Combination of ammonia and xylanase pretreatments: impact on enzymatic xylan and cellulose recovery from wheat straw. Bioresour Technol 101:6712–6717. doi:10.1016/j.biortech.2010.03.115. 20399643

[12] Rémond C, Boukari I, Chambat G, O’Donohue M. 2008. Action of a GH 51 alpha-l-arabinofuranosidase on wheat-derived arabinoxylans and arabino-xylooligosaccharides. Carbohydrate Polymers 72:424–430. doi:10.1016/j.carbpol.2007.09.008.

[13] Rakotoarivonina H, Hermant B, Aubry N, Rabenoelina F, Baillieul F, Remond C. 2014. Dynamic study of how the bacterial breakdown of plant cell walls allows the reconstitution of efficient hemicellulasic cocktails. Bioresour Technol 170:331–341. doi:10.1016/j.biortech.2014.07.097. 25151078

[14] Dupoiron S, Lameloise ML, Pommet M, Bennaceur O, Lewandowski R, Allais F, Teixeira ARS, Remond C, Rakotoarivonina H. 2017. A novel and integrative process: from enzymatic fractionation of wheat bran with a hemicellulasic cocktail to the recovery of ferulic acid by weak anion exchange resin. Ind Crops Prod 105:148–155. doi:10.1016/j.indcrop.2017.05.004.

[15] Husson E, Auxenfans T, Herbaut M, Baralle M, Lambertyn V, Rakotoarivonina H, Remond C, Sarazin C. 2018. Sequential and simultaneous strategies for biorefining of wheat straw using room temperature ionic liquids, xylanases and cellulases. Bioresour Technol 251:280–287. doi:10.1016/j.biortech.2017.12.047. 29288956

[16] Zerbino DR, Birney E. 2008. Velvet: algorithms for de novo short read assembly using de Bruijn graphs. Genome Res 18:821–829. doi:10.1101/gr.074492.107. 18349386 PMC2336801

[17] Bryson K, Loux V, Bossy R, Nicolas P, Chaillou S, van de Guchte M, Penaud S, Maguin E, Hoebeke M, Bessières P, Gibrat J-F. 2006. AGMIAL: implementing an annotation strategy for prokaryote genomes as a distributed system. Nucleic Acids Res 34:3533–3545. doi:10.1093/nar/gkl471. 16855290 PMC1524909

[18] Drula E, Garron ML, Dogan S, Lombard V, Henrissat B, Terrapon N. 2022. The carbohydrate-active enzyme database: functions and literature. Nucleic Acids Res 50:D571–D577. doi:10.1093/nar/gkab1045. 34850161 PMC8728194

